# Activation of c-Met and Upregulation of CD44 Expression Are Associated with the Metastatic Phenotype in the Colorectal Cancer Liver Metastasis Model

**DOI:** 10.1371/journal.pone.0097432

**Published:** 2014-05-13

**Authors:** Victoria A. Elliott, Piotr Rychahou, Yekaterina Y. Zaytseva, B. Mark Evers

**Affiliations:** 1 Markey Cancer Center, University of Kentucky, Lexington, Kentucky, United States of America; 2 Department of Surgery, University of Kentucky, Lexington, Kentucky, United States of America; Aix-Marseille University, France

## Abstract

**Background:**

Liver metastasis is the most common cause of death in patients with colorectal cancer. Despite extensive research into the biology of cancer progression, the molecular mechanisms that drive colorectal cancer metastasis are not well characterized.

**Methods:**

HT29 LM1, HT29 LM2, HT29 LM3 cell lines were derived from the human colorectal cancer cell line HT29 following multiple rounds of *in vivo* selection in immunodeficient mice.

**Results:**

CD44 expression, a transmembrane glycoprotein involved in cell-cell and cell-matrix adhesions, and cancer cells adhesion to endothelial cells was increased in all *in vivo* selected cell lines, with maximum CD44 expression and cancer cells adhesion to endothelial cells in the highly metastatic HT29 LM3 cell line. Activation of c-Met upon hepatocyte growth factor (HGF) stimulation in the *in vivo* selected cell lines is CD44 independent. *In vitro* separation of CD44 high and low expression cells from HT29 LM3 cell line with FACS sorting confirmed that c-Met activation is CD44 independent upon hepatocyte growth factor stimulation. Furthermore, *in vivo* evaluation of CD44 low and high expressing HT29 LM3 cells demonstrated no difference in liver metastasis penetrance.

**Conclusions:**

Taken together, our findings indicate that the aggressive metastatic phenotype of *in vivo* selected cell lines is associated with overexpression of CD44 and activation of c-MET. We demonstrate that c-Met activation is CD44 independent upon hepatocyte growth factor stimulation and confirm that CD44 expression in HT29 LM3 cell line is not responsible for the increase in metastatic penetrance in HT29 LM3 cell line.

## Introduction

Colorectal cancer (CRC) is the second leading cause of cancer-related deaths in the United States [1]. Metastatic or recurrent disease is the most common cause of death in these patients. The prognosis for CRC is based on the formation of distant metastases, not the primary tumor itself. Even with comprehensive research into the biology of cancer progression, the molecular mechanisms involved in the metastatic cascade are not well characterized.

The mechanisms of metastasis involve a selective and sequential series of steps, including separation from the primary tumor, invasion through surrounding tissues, entry into the circulatory system, and the establishment and proliferation in a distant location [2]. Two proteins that have been shown to be involved with multiple steps of the metastatic cascade are CD44 and c-MET. CD44, a transmembrane glycoprotein that belongs to a family of cell adhesion molecules, is involved with the progression and metastasis of multiple types of cancer [3]–[6] and has been associated with a poor prognosis in CRC patients [3]. c-MET is a proto-oncogene that encodes for the receptor tyrosine kinase, also known as hepatocyte growth factor receptor [4]. The only known ligand for c-MET is hepatocyte growth factor (HGF); both c-MET and HGF are upregulated in a number of malignancies and are associated with a poor prognosis and an early predictor of further metastasis [5]. Specifically, c-MET is involved in the regulation of proliferation, motility, invasion and metastasis via its phosphorylation and activation of downstream signaling pathways [4].

A comprehensive understanding of the mechanisms that drive CRC metastasis is important for the development of novel approaches to treat this cancer. Therefore, the purpose of our study was to identify the genes that promote liver metastasis in CRC. Here, we established three highly metastatic CRC cell lines and show that their more aggressive metastatic phenotype is associated with an increase in CD44 expression and activation of c-MET. Furthermore, we show that the activation of c-MET was independent of the levels of CD44 present. Finally, we demonstrate that increased CD44 expression is not responsible for the increase in metastatic penetrance the of HT29 LM3 cell line. Importantly, *in vivo* selection and isolation of liver-tropic CRC metastatic cells allowed us to study the biological mechanisms of CRC cancer metastasis and identify the mechanisms contributing to liver metastasis in CRC.

## Materials and Methods

### Cell Lines, Transfections

HT29 cells and Human Lung Microvascular Endothelial Cells (HMVEC-L) were obtained from American Type Culture Collection (Manassas, VA), were previously authenticated in November 2011 by Genetica DNA Laboratories (Cincinnati, OH) were cultured in McCoy’s 5A medium, purchased from Sigma Aldrich (St. Louis, MO) supplemented with 10% FBS and antibiotic-antimycotic. EGFP-N1 vector was purchased from Clontech (Mountain View, CA). GFP-expressing cells were selected with 500 µg/ml Geneticin (G418), purchased from Life Technologies (Carlsbad, CA) and enriched by three cycles of fluorescence-activated cell sorting (FACS). Pre-made pGL3 firefly luciferase (luc) lentiviral particles were purchased from Lentigen (Gaithersburg, MD). For lentiviral transduction, 5000 cells/well were seeded in 96 well tissue culture plates and infected the following day with luc lentiviral particles at a MOI of 10 in the presence of 10 µg/ml polybrene, purchased from Santa-Cruz Biotechnology (Dallas, TX).

### Liver Metastasis Model and *in* *vivo* Imaging

Male athymic NCr nude mice between 6–8 wks of age were purchased from Taconic (Hudson, NY). Housing for these animals was maintained in a HEPA-filtrated environment within sterilized cages with 12 h light/12 h dark cycles. All animal procedures were conducted with approval of and in compliance with University of Kentucky Institutional Animal Care and Use Committee; protocol #2009-0529. For intrasplenic injection of CRC cells athymic NCr nude mice were anesthetized with isoflurane (induction 4%, maintenance 2%). A 1 cm cutaneous incision was made in the left flank and carried down through the peritoneal wall. The spleen was carefully exposed, and HT29 GFP-Luc cells (5×10^6^ cells/100 µl) were injected under the spleen capsule using a 27-gauge needle. The viability of cells used for inoculation was greater than 95% as determined by Vi-CELL XR (Beckman Coulter). Gentle pressure was applied to the inoculation site until there was no visible sign of bleeding. LIGACLIP extra single clip ligating applier with titanium LIGACLIP extra ligating clips, purchased from Ethicon (San Angelo, TX), was used to clip lienal and lieno-pancreatic veins 5 min after splenic injection of CRC cells; the spleen was then removed. The mice were sacrificed after 4 wks or sooner if moribund. Liver tissues were preserved for histological examination by fixation in 10% buffered formalin followed by paraffin embedding.

All bioluminescent images were acquired with an IVIS Spectrum (Caliper Life Sciences, Hopkinton, MA), with the stage heated to 37°C during live cell imaging. Images were acquired 10 min after i.p. injection of D-Luciferin (150 mg/kg) using 15 sec exposure. GFP fluorescence imaging was performed using an LT-9500 Illumatool/TLS (Lightools Research, Encinitas, CA), equipped with an excitation source (470 nm) and filter plate (515 nm).

### Enzymatic Isolation of CRC Cells from Liver Metastases

Liberase DH Research Grade (05401054001; Roche Applied Science) was resuspended in sterile water to 2.5 mg/ml concentration and stored in single-use 100 µl aliquots at−80°C. Collagenase/Hyaluronidase (07912; StemCell Technologies) was aliquoted into single-use 250 µl aliquots and stored at −80°C. Upon collection, metastatic tumors were placed into complete cell culture media supplemented with 1X Gibco Antibiotic-Antimycotic (15240-062; Life Technologies) for transportation. Metastatic tumor fragments were minced into 2-mm cubes using scissors and digested in 50 µg/ml Liberase DH (100 µl) and 0.5X Collagenase/Hyaluronidase (250 µl), diluted in 5 mL of McCoy5A serum free media for 4 h at 37°C with gentle agitation by magnetic stirring bar. No undigested tissue was observed. Digested cells were washed twice with complete cell culture media and transferred into 10% FBS McCoy5A media supplemented with 1X Gibco Antibiotic-Antimycotic and 100 µg/ml Primocin (ant-pm-1; InvivoGen).

### Western Blot Analysis and Antibodies

Total protein lysates (20 µg) were resolved on a 4–12% bis-tris gel and transferred to Immobilon PVDF transfer membranes. Membranes were incubated for 40 min at room temperature in blocking solution (TRIS-buffered saline containing 5% nonfat dried milk and 0.1% Tween 20), followed by an overnight incubation in primary antibodies at 4°C. Membranes were then washed 3 times and incubated with horseradish peroxidase–conjugated secondary antibodies for 1 h. After 3 additional washes, the immune complexes on the membranes were visualized by ECL detection.

The following antibodies were purchased and utilized in our study: Cell Signaling (Danvers, MA): phospho-AKT (#4058), total AKT (#2920), AKT2 (#3063), phospho-ERK 1/2 (#4695), total ERK ½ (#9107), PTEN (#9559), phospho-mTOR (#2971), total mTOR (#2972), phospho-MET (#3129 for western blotting and #3077 for IHC), c-MET (#3148), CD44 (#3570), phospho-beta catenin (#4176), phospho-FAK (#8556), and total FAK (#3285). Santa Cruz (Dallas, TX): AKT 1 (#5298). BD (San Jose, California): Beta catenin (#610154) and E-cadherin (#610404). Abcam (Cambridge, MA): KRAS (#55391). Millipore (Billerica, MA): p85α (#05-212). All antibodies were used at a concentration of 1∶1,000.

### siRNA Transfections and HGF Cell Treatment

For HGF, treatment cells were seeded at a concentration of 800,000 cells/well in a six well plate. After 24 h, the cell media was changed to serum free media for an additional 24 h. Recombinant human HGF (PeproTech #100-39) was then added to the wells at a concentration of 50 ng/mL for 5 min. siRNA transfections: ON-TARGET plus CD44 siRNAs (LU-00999907, LU-00999908) and control siRNA (D-001810-10) were purchased from Dharmacon (Lafayette, CO) and used in a concentration of 100 µM.

### Immunohistochemistry

Immunohistochemistry (IHC) was performed as previously described [6]. The paraffin-sections were deparaffinized in xylene and rehydrated in descending ethanol series. Protein staining was performed using DAKO EnVision Kit, purchased from Dako Corp. (Carpinteria, CA). CD44 and p-MET antibodies were used at a concentration of 1∶100 in DAKO antibody diluent. All sections were counterstained with hematoxylin and observed by light microscopy.

### Proliferation Assay

Parental HT29 and HT29 LM3 cells were plated in 24-well plates at a density of 25,000 cells per well. Proliferation was assessed by cell counting at 24, 48, and 72 h, using a Beckman-Coulter Vi Cell XR cell viability analyzer (Fullerton, CA).

### Endothelial Cell Adhesion Assay

Human microvascular endothelial cells from lungs (HMVEC-L) were activated with 15 ng/mL of TNFα for 4 h. Parental HT29 and HT29 LM3 cells were labeled with Calcein AM (2.5 mg/mL final concentration) for 30 min at 37°C, washed, and added atop the monolayer of activated HMVEC-L cells for 30 min. Unattached cells were removed by washing with PBS (5x) and three GFP images were taken per well to count the attached cells.

### Flow Cytometry Analysis and Cell Sorting

HT29 LM3 cells were labeled with CD44− Alexa Fluor 647 antibody (Biolegend) at a concentration of 2.5 µg/mL per 10^7^ cells for 30 min. The cells were washed and resuspended in sorting buffer (1X phosphate buffered saline, 1 mM EDTA, 25 mM HEPES, 1% heat-inactivated fetal bovine serum). Within a sample of HT29 LM3 cells, we sorted two populations of cells: 10% of the cells with a high expression of CD44 (abbreviated as HT29 LM3 CD44+) and cells that do not express CD44 (abbreviated as HT29 LM3 CD44−). Unstained HT29 LM3 cells were used as a negative control. Following cell sorting, the cells were expanded in cell culture. The UK Flow Cytometry Service Facility conducted cell analysis and cell sorting.

## Results

### *In vivo* Selection Process of Liver-tropic CRC Metastatic Cells

CRC liver metastasis is a multistage process in which malignant cells spread from a primary tumor to colonize the liver. Malignant cells are invasive and metastatic; however, only a restricted fraction of the cells in a primary tumor are considered to be highly metastatic. *In vivo* selection methods, using primary cancer cell lines and comparison between pure clonal populations of isolated liver-tropic metastatic cells is a useful scientific approach for the identification of biological mechanisms enhanced during CRC liver metastasis.

**Figure 1:**
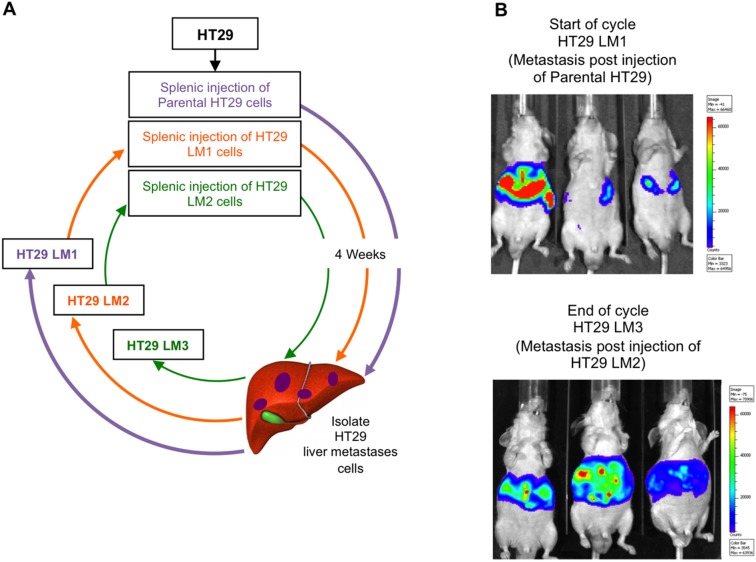
Establishment of HT29 derived cell lines with enhanced metastatic potential. (**A**) Illustration of metastatic CRC *in* *vivo* selection process. Experimental liver metastases were harvested, established in culture and designated HT29 LM1, LM2, and LM3 cell lines. (**B**) Bioluminescent images of mice 4 wks after intrasplenic injection of parental HT29 and HT29 LM2 cell lines.

In this study, we engineered the HT29 CRC cell line to express reporter plasmids, GFP and firefly luciferase that permits fluorescence and bioluminescence imaging in a single experimental model, and performed *in* *vivo* selection of HT29 cells that metastasized to the liver. Briefly, HT29 cells were injected into the spleen of athymic nude mice; splenectomy was performed 5 min after intrasplenic injection of CRC cells to avoid re-metastasis. Experimental liver metastases (LM; 30–40 metastatic lesions) were harvested, established in tissue culture, and designated as the HT29 LM cell lines. Cells harvested from these cultures were injected into the spleen of another set of nude mice. The sequence of *in* *vivo* selection is shown in **Figure 1A**. HT29LM1 and HT29 LM3 vary dramatically with respect to their metastatic potential. Bioluminescent imaging of mice injected with HT29 LM2 demonstrated a 2.5-fold increase in penetrance compared to parental cell line (∼3.5 wks with ∼100% penetrance vs. ∼4 wks with ∼40% penetrance) (**Figure 1B**). Thus, *in* *vivo* selection of HT29 cell lines provided highly metastatic cells for comparison to parental cells under isogenic background.

### The c-MET Pathway is Upregulated in the HT29 Derived Cell Lines

**Figure 2:**
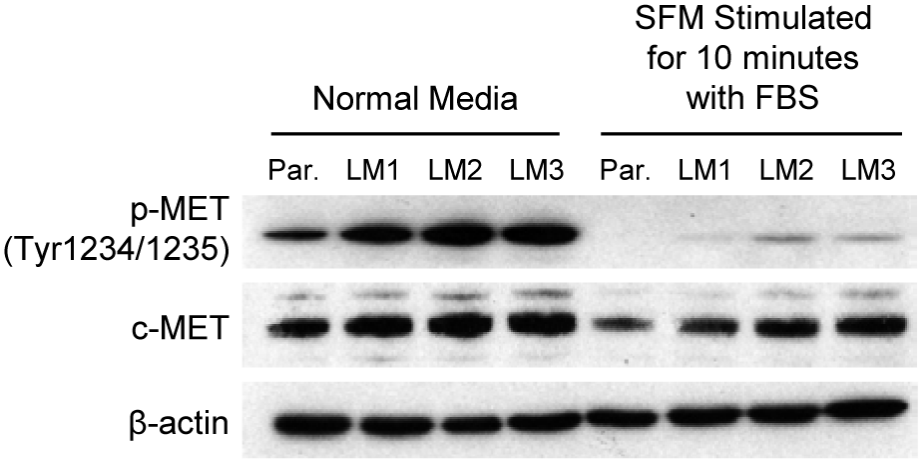
The aggressive metastatic phenotype of the HT29 LM1, LM2, and LM3 cell lines is associated with the activation of the c-MET pathway. HT29 LM1, LM2, LM3 and parental cell line were cultured in normal medium for 24(first 4 lanes). In another experiment, cells were cultured in serum free medium for 24 h and stimulated with complete medium for 10 min (remaining 4 lanes). Protein expression profiles were analyzed by Western blot. β-actin was used as a loading control.

Molecular analysis of cancer cells in various stages of progression have revealed that alterations in tumor suppressor genes and oncogenes accumulate during tumor progression and correlate with the clinical aggressiveness of cancer. Next, we performed comparative analysis of several oncogenes and tumor suppressor gene expression profiles in HT29 LM cell lines by Western blot. **Figure 2** demonstrates that the phosphorylation of c-MET was dramatically increased in the HT29 LM1 and HT29 LM2 cell lines, with the highest activation in the HT29 LM3 cell line, as compared to the parental HT29 cells. We observed a slight increase in total c-MET protein as well. These changes were noted when cells were cultured in normal or serum-free conditions (separate experiments). Western blot analysis of protein extracts from HT29 LM cell lines did not show any changes in pAkt (Ser473), Akt, p85α, PTEN, pERK(Tyr 1234/1235), ERK, k-Ras protein expression (data not shown). Together, these findings suggest that an increase in the metastatic potential of HT29 derived cell lines is associated with activation of the c-MET pathway.

### Expression of CD44 Protein is Significantly Increased in the HT29 Derived Cell Lines

**Figure 3:**
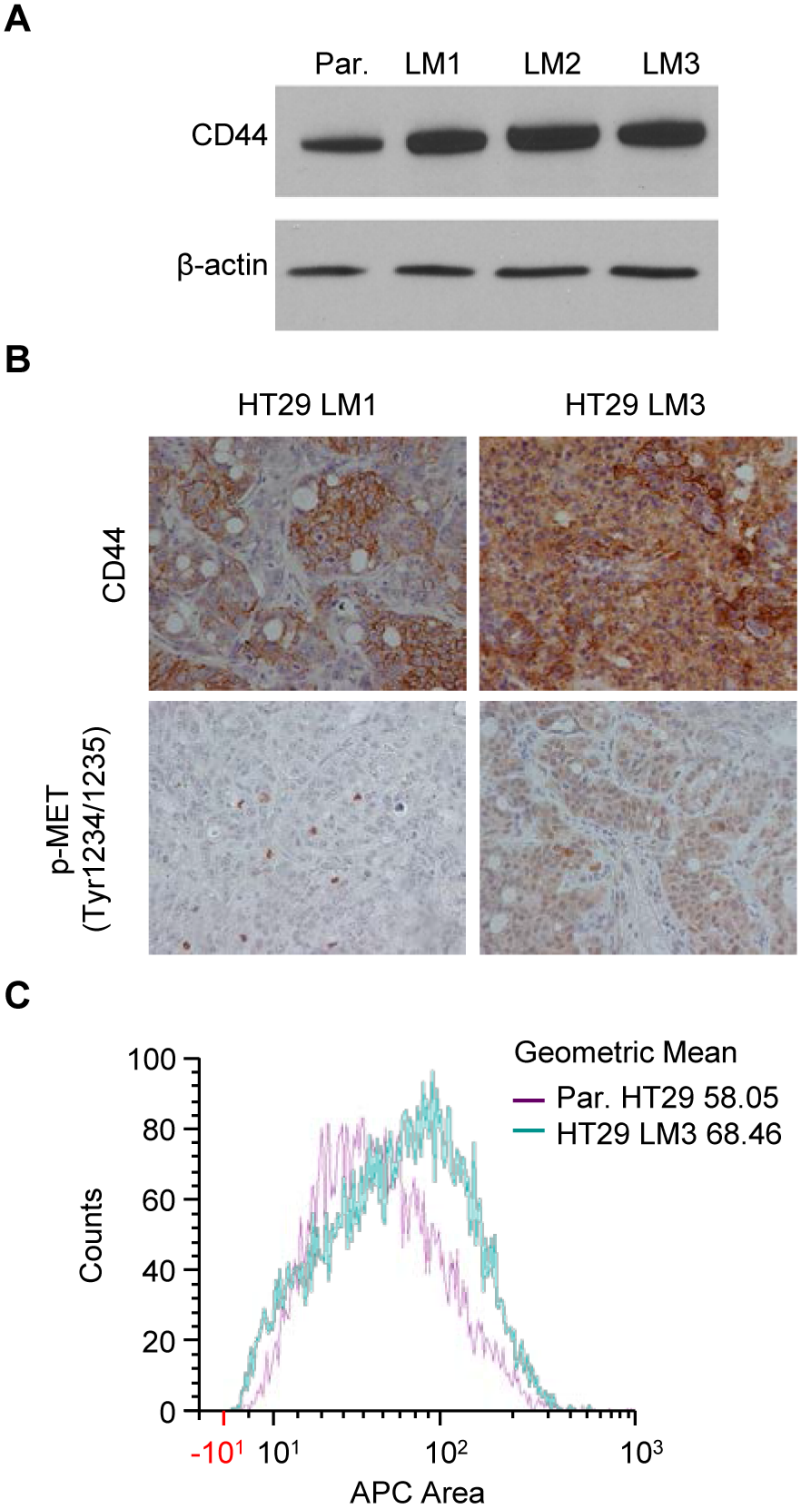
Expression of CD44 is significantly increased in the HT29 LM1, LM2, and LM3 cell lines. (**A**) HT29 LM1, LM2, LM3 and parental cell line were cultured in normal medium for 24 h. Protein expression profiles were analyzed by Western blot. β-actin was used as a loading control. (**B**) IHC analysis of CD44 and p-MET expression in HT29 LM1 and HT29 LM3 liver metastasis tissue sections. (**C**) Flow cytometry analysis of CD44 geometric mean fluorescence intensity in parental HT29 and HT29 LM3 cells.

The expression of key proteins in the CD44, β-catenin, and FAK pathways, which play an important role in CRC metastasis [7]–[9] was analyzed next. We observed a gradual increase of high molecular weight CD44 expression observed at ∼150 kDa in HT29 LM cell lines, with maximum CD44 expression in HT29 LM3 compared to the parental HT29 cell line (**Figure 3A** **)**. Western blot analysis of protein extracts from HT29 LM cell lines did not show any changes in p-β-Catenin (S675), β-Catenin, p-FAK(Tyr397) and FAK protein expression (data not shown). To further confirm our *in* *vitro* findings, we analyzed parental HT29 and HT29 LM3 liver metastasis tissue sections by IHC. Consistent with Western blot analysis, expression of both p-MET and CD44 was significantly increased in HT29 LM3 as compared to HT29 LM1 (**Figure 3B**). CD44 cell surface flow cytometry analysis also demonstrated higher level of CD44 expression on the surface of HT29 LM3 cells compared to parental HT29 cells (**Figure 3C** **)**. Together, these data suggest that enhanced metastatic potential of the HT29 LM cell lines is associated with an increase in CD44 expression.

### High Level of c-MET Activation in HT29 LM3 is Independent of CD44 Expression

**Figure 4:**
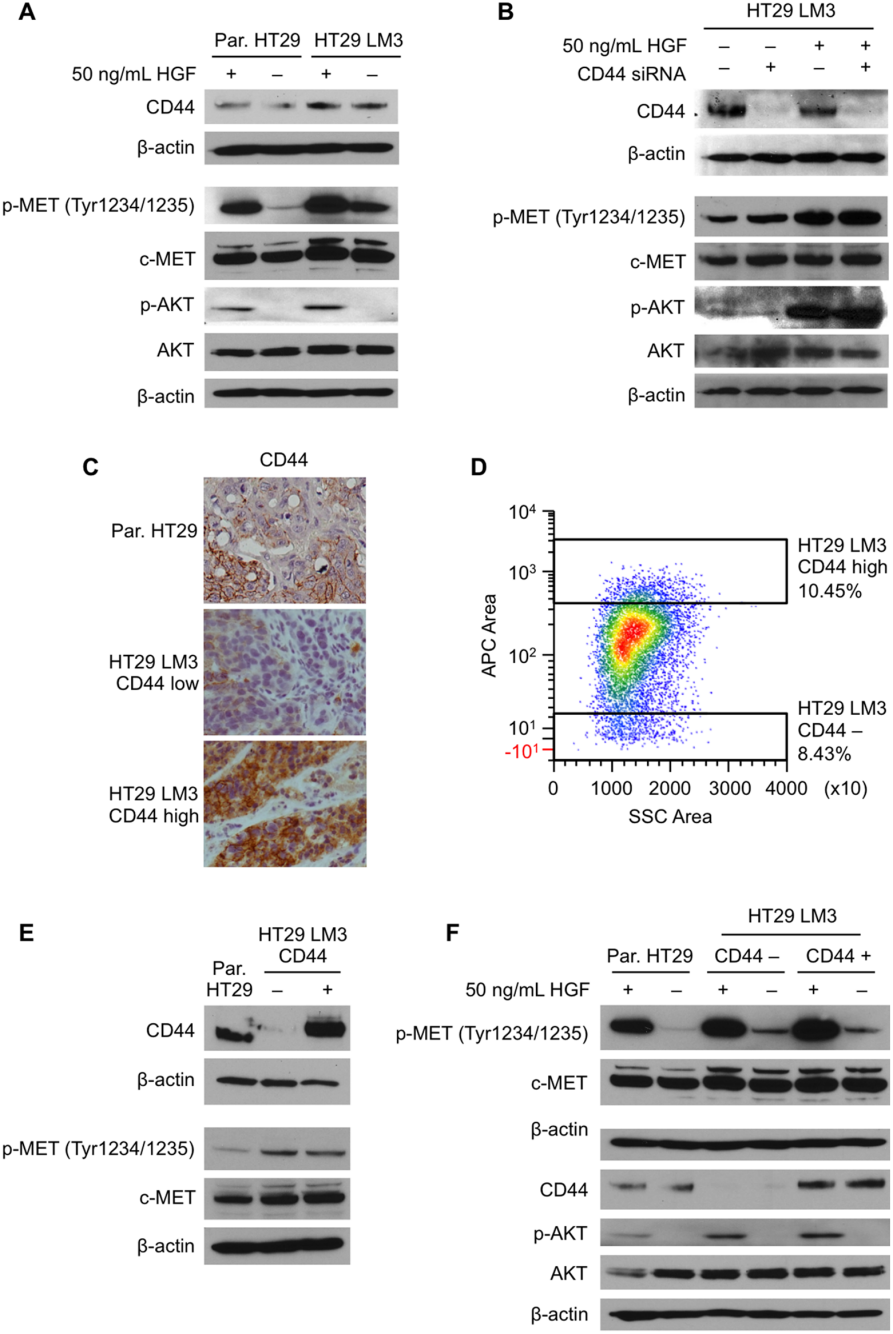
CD44 independent c-MET activation in HT29 LM3 cells. (**A**) Analysis of CD44, p-MET, c-MET, p-AKT and AKT expression in parental HT29 and HT29 LM3 after HGF stimulation (50 ng/mL; 5 min) by Western blot. β-actin was used as a loading control. (**B**) HT29 LM3 cells were transfected with CD44 siRNA and stimulated with HGF (50 ng/mL; 5 min) 24 h after siRNA transfection. (**C**) CD44 expression in HT29 LM3 and parental HT29 liver metastasis tissue sections were analyzed by IHC. (**D**) HT29 LM3 cells were sorted for high and low CD44 expression. Cells were gated and sorted to collect 10% of the cells with high CD44 expression and cells with low CD44 expression. (**E**) Analysis of CD44, p-MET, and c-MET expression in parental HT29, HT29 LM3 CD44−, and HT29 LM3 CD44+ cells. (**F**) Analysis of p-MET, c-MET, CD44, p-AKT, and AKT expression in parental HT29, HT29 LM3 CD44−, and HT29 LM3 CD44+ cells after HGF (50 ng/mL; 5 min) stimulation.

Met is an essential receptor tyrosine kinase (RTK) that induces cancer cells proliferation, differentiation, migration and survival [10]. Met is transiently activated after HGF induction and requires specific CD44 isoforms for its activation in various cancers [11], [12]. To better understand the interaction between MET activation and CD44 in the parental HT29 and HT29 LM3 cells, we analyzed the effects of HGF stimulation in both cell lines. As shown in **Figure 4A**, HGF-treatment increases activation of c-MET in both parental HT29 and HT29 LM3, with slightly higher activation in the HT29 LM3 cell line as compared to the parental HT29 cell line. We next transfected HT29 LM3 cells with siRNA against all CD44 isoforms and then treated these cells with HGF. **Figure 4B** demonstrates similar levels of c-MET phosphorylation in the presence or absence of HGF, suggesting that activation of the c-MET pathway in HT29 LM3 cells is independent of CD44 expression.

IHC analysis of CD44 in HT29 LM3 tissue sections demonstrated variable CD44 expression in liver metastases; clusters of liver metastases with high expression of CD44 next to metastases with low CD44 expression (**Figure 4C**). To further analyze this phenomenon, we used flow cytometry and cell sorting to isolate two populations of cells based on cell surface CD44 expression. Two cell lines with high CD44 expression (HT29 LM3 CD44+) and low CD44 expression (HT29 LM3 CD44−) were established (**Figure 4D**). CD44 expression in these cell lines was confirmed by Western blot analysis. Both CD44+ and CD44− cell lines had higher activation of c-Met compared to the parental cell line (**Figure 4E**).

To further confirm that activation of c-MET is independent of CD44 expression in the HT29 LM3 cell line, CD44+ and CD44− cell populations were stimulated with HGF and analyzed by Western blot. As shown in **Figure 4F**, a similar level of c-MET activation in CD44+ and CD44− cells was observed. Therefore, we show that the level of CD44 expression in *in* *vivo* trained HT29 LM3 cells does not correlate with c-Met activation levels by its natural ligand, HGF, further confirming that c-MET acts independently of CD44 in the HT29 LM3 cell line.

### Aggressive Metastatic Behavior of HT29 LM3 is Associated with an Increased Ability to Adhere to Endothelial Cells but is not Driven by CD44 Expression

**Figure 5:**
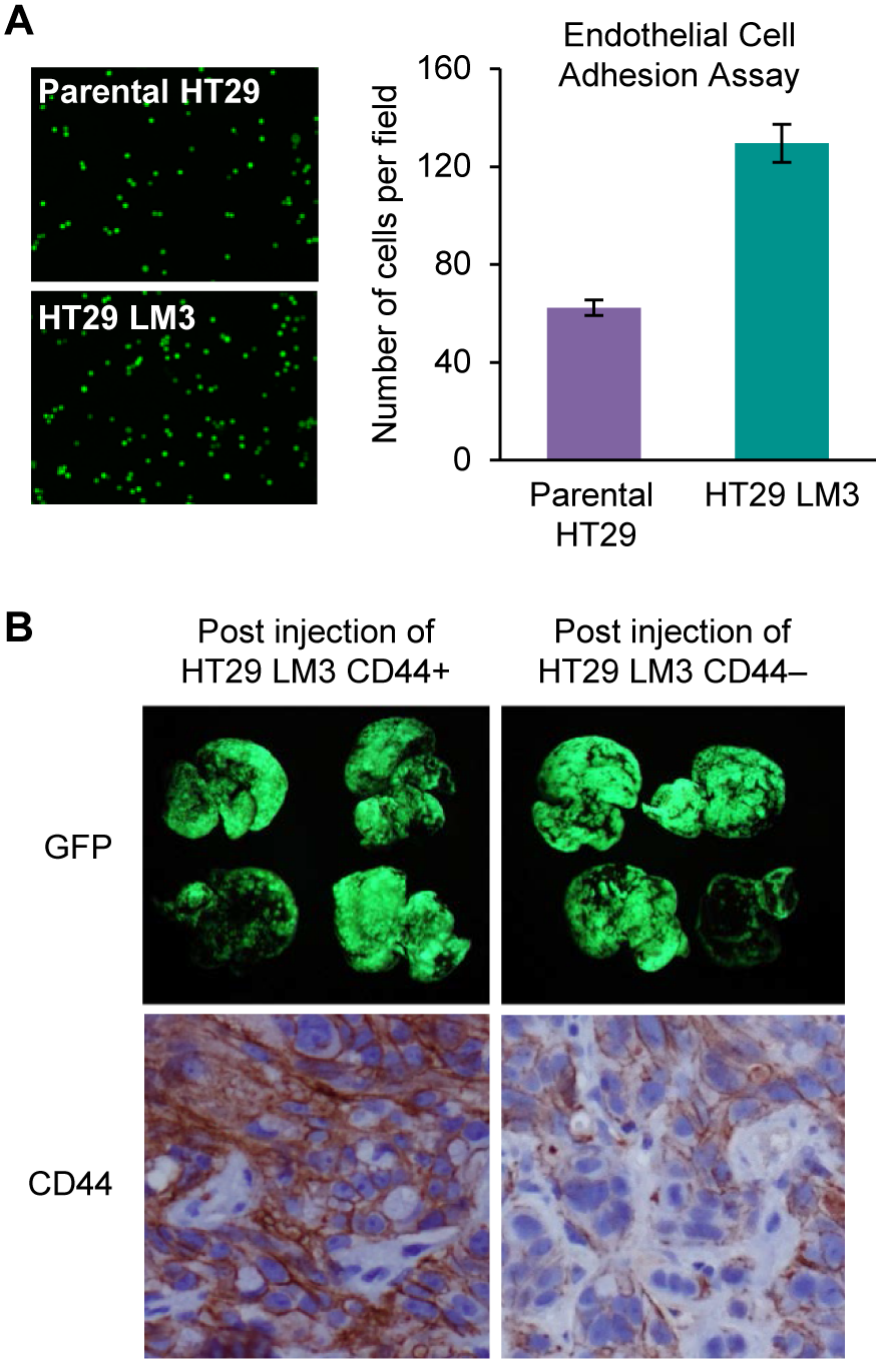
Role of CD44 high and CD44 low expression in CRC liver metastasis. (A) Adhesion of parental HT29 and HT29 LM3 cells to HMVEC-L cells was assessed as described in Materials and Methods. Data shown as mean fold changes in number of parental HT29 cells attached to HMVEC-L cells versus HT29 LM3 cells (*p<0.001). Representative images show adhesion of parental HT29 and HT29 LM3 cells to HMVEC-L cells. (B) Fluorescent GFP imaging of liver metastasis 4 wks after intrasplenic injection of HT29 LM3 CD44+ and HT29 LM3 CD44− cells (5×10^6^; 100 µl of PBS). IHC analysis of CD44 expression in CD44 high and CD44 low CRC liver metastasis.

Cancer cell adhesion to endothelial cells is an important step of metastasis and is known to be regulated by CD44 in many cancer cells [13]. HT29 LM3 and parent HT29 cell lines were labeled with Calcein AM and HT29 LM3 binding ability to endothelial cells was evaluated with *in* *vitro* adhesion assay. We demonstrated that HT29 LM3 cells had an increased ability to attach to endothelial cells, as compared to parental HT29 cells (**Figure 5A**). These results suggest that the more aggressive metastatic phenotype of HT29 LM3 cells is may be the result of increased cellular adhesion to endothelial cells.

Our *in* *vitro* analysis of *in* *vivo* selected HT29 cell lines identified an increase in CD44 expression and c-Met activation. Since adhesion to endothelial cells is a first crucial rate-limiting step of hematogenous CRC liver metastasis after intrasplenic injection and the CD44 receptor is known to regulate cancer cells adhesion [7], [12], we next evaluated role of CD44 on CRC metastasis *in* *vivo*. HT29 LM3 CD44+ and HT29 LM3 CD44− cells were injected into the spleens of athymic nude mice. Four wks post injection, fluorescent GFP imaging of CRC liver metastases was performed. As shown in **Figure 5B**, the level of CD44 expression did not have any significant effect on CRC liver metastasis. CD44 expression level in CD44+ and CD44− liver metastases was confirmed with IHC staining. Collectively, these findings suggest that overexpression of CD44 alone is not sufficient to enhance CRC liver metastasis and that other pathways are likely involved.

## Discussion

The most devastating aspect of CRC is the emergence of liver metastases, which is responsible for the majority of deaths from this disease. Thus, to understand the molecular mechanisms of metastasis is one of the most important issues in cancer research. According to the concept of tumor cell heterogeneity, highly metastatic cells are present as a sub-population in a primary tumor [14]. At present, it is impossible identify metastatic and non-metastatic cells in the primary tumor. In this study, we utilized an *in* *vivo* selection model to identify molecular markers for the prediction of metastatic potential of CRC cells. This model of injecting cancer cells into the spleen, harvesting hepatic metastases, and re-injecting into the spleen, creates highly metastatic cell lines as confirmed by a greater number of lymph and liver metastases [15]. Similarly, our study demonstrated that the cells created through *in* *vivo* selection cycle yielded extensive liver metastasis and that aggressive behavior of these cells is associated with alterations in CD44 expression, c-MET activity and increased ability of CRC cells to adhere to endothelial cells. Therefore, the model of *in* *vivo* selection for metastatic cells can be a useful tool for studying genetic changes occurring in cells when they acquire the metastatic phenotype. Furthermore, the utilization of this model allows for a better understanding of the mechanisms that drive cancer progression and can be used as a tool to discover and develop potential therapeutic targets for CRC metastasis.

c-MET and CD44 are co-expressed in a number of cancers, such as pancreatic cancer and CRC [16], [17]. Pancreatic cancer cells with a high expression of both c-MET and CD44 were shown to have a greater tumorigenic potential [17]. Furthermore, expression of both proteins have been correlated with a shorter patient survival period in CRC [3], and a recent study from our laboratory showed that CD44 and c-MET activation is associated with an increase in CRC metastasis [18]. Consistent with these findings, the results of our current study demonstrate that an increase in both the expression of CD44 and the activation of c-MET correlates with an increase in the metastatic potential of the HT29 LM3 cell line. CD44 and c-MET collaboration, and their interactions on the plasma membrane, lead to the activation of downstream signaling pathways that promote cancer progression [19]–[21]. On the other hand, several studies have found that the activation of c-MET is independent of CD44 [22], [23]. Our results suggest that in the HT29 LM3 cell line, CD44 and c-MET act independently in the presence or absence of HGF, a c-MET ligand. Discrepancies between studies could be explained by differences in the cell lines used and indicate that the extent of the interaction between CD44 and c-MET may be cell type specific. As both proteins have been associated with a poor prognosis in a multitude of cancers, the further study of their interaction in metastatic cell lines is critically important and may lead to new therapeutic targets.

To better understand the role of CD44, we utilized two populations of HT29 derived cells, CD44+ and CD44−. Interestingly, our data suggests that CD44 is not the key protein driving the HT29 derived cells to become increasingly metastatic and that most likely, c-Met activation or other pathways enhance CRC liver metastasis. In our model, CD44 and c-MET signal independently, which indicates that c-MET signaling is not impaired in CD44− cells and continues to drive metastasis. Additionally, there are many isoforms of CD44, due to alternative splicing, that play different roles in metastasis, which may explain the discrepancy between the roles of CD44 in metastasis. Each isoform plays a different role in cancer cells and some cancers only express one of the isoforms while others express multiple isoforms [7]. Therefore determining the exact function that CD44 plays in each cancer cell line becomes increasingly important for the utilization of CD44 as a therapeutic target.

## Conclusions

A thorough understanding of the genetic mechanisms that initiate the metastatic cascade and promote CRC metastasis progression in the liver is important for the development of novel anticancer therapies. Pure clonal populations of isolated liver-tropic metastatic cells allowed us to selectively study and compare biological mechanisms mediating CRC metastasis to liver. Taken together, our findings indicate that the aggressive metastatic phenotype of the *in* *vivo* selected HT29 cell line is associated with overexpression of CD44 and activation of c-MET. We demonstrate that c-Met activation is CD44 independent upon HGF stimulation and confirm that CD44 expression in HT29 LM3 cell line is not responsible for the increase in metastatic penetrance in HT29 LM3 cell line. Importantly, we demonstrate that the model of *in* *vivo* selection for CRC metastatic cells represents a valuable tool to identify the mechanisms contributing to liver metastasis in CRC and identify molecular pathways for a targeted therapy of CRC liver metastasis.
